# Editorial: Diversity of beetles and associated microorganisms, volume II

**DOI:** 10.3389/fmicb.2026.1805361

**Published:** 2026-03-11

**Authors:** Takema Fukatsu, Minoru Moriyama, Kohei Oguchi, Hisashi Kajimura, Antonio Gugliuzzo

**Affiliations:** 1 Molecular Biosystems Research Institute, National Institute of Advanced Industrial Science and Technology (AIST), Tsukuba, Japan; 2 Department of Biological Sciences, The University of Tokyo, Tokyo, Japan; 3 Graduate School of Life and Environmental Sciences, University of Tsukuba, Tsukuba, Japan; 4 Misaki Marine Biological Station, The University of Tokyo, Miura, Japan; 5 Graduate School of Bioagricultural Sciences, Nagoya University, Nagoya, Japan; 6 Department of Agriculture, Food and Environment, University of Catania, Catania, Italy

**Keywords:** bacteria, beetles, Coleoptera, fungi, insects, microbial interactions, mutualism, symbiont

Beetles (Insecta: Coleoptera) comprise the most diverse macroscopic organismal group in the terrestrial ecosystem, which embrace over 400,000 described species, and nobody knows how many beetle species actually exist (Grimaldi and Engel, 2005; McKenna et al., 2019). On the other hand, microbiologists recognize that the “real” extent of biodiversity resides in microbial communities (Thompson et al., 2017; Louca et al., 2019). Coleopterans serve as a key example of this phenomenon, harboring complex microbial consortia across a vast array of ecological niches. In fact, different beetles host different microorganisms, encompassing bacteria, fungi, protists, viruses, and others in their gut, body cavity and/or cells, or on their body surface and ambient environments (Salem et al., 2015; Hosokawa and Fukatsu, 2020). Reflecting the diversity of both the hosts and the microbes, their associations are ubiquitous and diverse.

Following the success of the previous Research Topic “*Diversity of beetles and associated microorganisms*” (Salem et al., 2023), this second volume is established, which provides an opportunity to collect and overview recent achievements emerging in this research field with an extended coverage. In total, eight original research articles, two review articles and one brief research report are compiled here. These contributions cover microbial associates across a diverse range of taxa, including ladybird beetles (Coccinellidae), bark and ambrosia beetles (Platypodinae and Scolytinae, Curculionidae), a weevil (Curculionidae), a scarab beetle (Scarabaeidae) and a flour beetle (Tenebrionidae).

Many coccinellid species, also known as ladybirds or lady beetles, are predators actively preying on small insects, some of which have been utilized as biological control agents against aphids, whiteflies, scale insects, and other notorious agricultural pests (Obrycki and Kring, 1998). Therefore, not only in basic biology but also in applied science, it is important to understand how microbial associates affect physiological and ecological traits of their coccinellid hosts. The multi-colored Asian lady beetle, *Harmonia axyridis*, is one of the most famous coccinellid species for its conspicuous color and behavior, its extreme color variation, its worldwide invasive problems, and its utilization as a pest control agent (Koch, 2003). Sun et al. observed that cold-stored *H. axyridis* exhibited a great decrease in egg hatch rate during later oviposition period, and furthermore, their F1 offspring suffered complete loss of hatchability. When cold-stored female beetles and control non-stored female beetles, and also their F1 eggs, were examined for their microbiomes, some differences were detected between the treatment groups. The discolored lady beetle, *Micraspis discolor*, is found in wetland environments including rice fields, preys on various rice pests such as aphids, planthoppers, leafhoppers, thrips, etc., and is considered as a potential biocontrol agent (Rattanapun, 2012). Notably, it was reported that *M. discolor* feeds not only insects but also plant pollen—it tends to be found on flowering rice plants and feeds on rice pollen, uncovering that it is not purely carnivorous (Shanker et al., 2013). Li et al. observed that antibiotic treatment impairs the performance of the insects fed with pollen but does not affect the performance of the insects fed with moth eggs, suggesting that gut bacteria may be involved in pollen feeding capability of the host beetles. The authors identified a strain of *Serratia marcescens* as a specific gut bacterium of *M. discolor* and showed that oral reintroduction of *S. marcescens* partially restored the performance of the antibiotic-treated insects even when they were fed with pollen. These studies provide basic information on microbial associates of the coccinellid beetles and their potential effects on physiology and performance of the host insects that are regarded as promising biocontrol agents.

Bark and ambrosia beetles represent key drivers of ecosystem dynamics, relying on sophisticated multi-trophic mutualisms with diverse microbial consortia. These symbioses range from the facultative phoretic associations in bark beetles, which aid in the detoxification of host-tree defenses, to the obligate “fungiculture” of ambrosia beetles, where species-specific fungi serve as the unique nutritional source (Biedermann and Vega, 2020). Beyond these well-documented roles, there is a vast array of still-unexplored microbial interactions within this ecological group, which requires often deeper investigation (Costanzo et al., 2026). Consequently, there is an urgent need for novel studies aimed at deciphering the functional complexity of these cryptic associations to better predict and manage the ecological impacts of beetle-microorganism associations in a changing world (Hulcr et al., 2020).

In this context, the study by Tanin et al. investigated the behavioral responses of the European fir bark beetles, *Pityokteines curvidens* and *P. vorontzowi*, to their core fungal associates. In particular, utilizing a novel two-tier bioassay, the authors observed a stronger preference by beetles for certain fungi such as *Geosmithia* sp. and *Ophiostoma piceae* through physical contact rather than volatiles alone. Interestingly, beetle females responded to volatile cues, while males did not. These findings suggest that bark beetle-fungus interactions are not merely physiological but are deeply embedded in the beetle social and reproductive ecology, supporting previous observations about the crucial role of fungal symbiont volatiles on bark and ambrosia beetle behavioral traits (Gugliuzzo et al., 2023; Kandasamy et al., 2023).

The study by Decker et al. focused on host specialization of fungal symbionts of the oak pinhole borer, *Platypus cylindrus*. The authors investigated the growth dynamics of the beetle primary nutritional symbiont, *Dryadomyces montetyi*, across different tree species via fungal culturing on media infused with extracts from both host and non-host trees. Significant variations were found in fungal biomass and expansion rates, which were higher for deciduous host trees, suggesting that the polyphagy typically attributed to ambrosia beetles could be constrained by the host-related specialization of their fungal mutualists, even if other generalist species have previously shown less specific adaption to semi-artificial media mimicking either a coniferous or a broadleaved host-tree species (Melet and Biedermann, 2024).

Popa et al. conducted a comprehensive meta-analysis spanning over 80 years of literature about *Ips typographus* mycobiome research. In particular, their review article synthesizes the diversity of fungal communities associated with the Eurasian spruce bark beetle. Analyzing 58 studies, they document 712 fungal species, identifying a “core mycobiome” of 14 consistently recorded phytopathogens. The study highlights a geographical imbalance in research, with a lack of data from the beetle's Asian range, and discusses the shift from culture-dependent to high-throughput sequencing methods. This review establishes a crucial foundation for understanding how the mycobiome can facilitates the ecological dominance of *I. typographus* in spruce forests.

The review article by Cambronero-Heinrichs et al. highlights while fungal associates have traditionally dominated the study of ambrosia beetles, their bacterial communities have been less explored. In particular, a comprehensive synthesis of current knowledge from metabarcoding studies, revealing that bacterial communities are often species-specific and differ between the beetles and their fungal gardens, is provided by the authors. The review identifies critical gaps, particularly the functional roles of dominant taxa in nitrogen fixation and detoxification. By proposing a roadmap for future research using meta-genomics, this work sets the stage for a more holistic approach when studying ambrosia beetle ecology.

Cruz et al. investigated the functional roles of multiple fungal associates of the tea shot hole borer (TSHB), *Euwallacea perbrevis*, in Florida. Through larval feeding assays and pathogenicity tests, the authors identified *Fusarium* sp. FL-1 and AF-8 as the primary nutritional symbionts, which also serve as the main drivers of *Fusarium* dieback in avocado trees. The study demonstrates that these two fungi are highly persistent in the mycangia of beetles even when other fungi co-occur in the substrate. Outcomes of this research are essential to develop novel management strategies targeting both the beetle vector and associated phytopathogens, as recently reported for similar targets (Costanzo et al., 2025a,b; Pan et al., 2025).

Understanding the microbiome of cryptic pests is vital, but correctly fine-tuning the experimental setup of such a kind of investigations can be challenging. In the study by Maurin et al., the authors address the critical challenge of determining the minimal sampling effort when studying the microbiome composition of the white pine cone beetle, *Conophthorus coniperda*. The research investigated how the number of insect guts per composite sample and the number of trees can impact microbiome recovery. Findings revealed little effect of the number of beetle guts, but highly significant effect of tree-to-tree spatial heterogeneity. Statistical models suggest that sampling approximately 33 trees per hectare is required to capture more than 95% of the local microbial diversity. These results emphasize that landscape-scale sampling is more critical for accuracy than increasing the number of individuals per pool, supporting the importance of spatial-variability when investigating the microbiome of cryptic beetle species (Hulcr et al., 2025; Six et al., 2026).

The citrus root weevil *Diaprepes abbreviatus* is known as a devastating agricultural pest infesting hundreds of crop plants that poses significant challenges to pest management (Simpson et al., 1996). Rodriguez-Fernandez et al. examined intestinal morphology, pH, and microbiota of adult females and males of *D. abbreviates*, which identified several representative bacterial taxa such as *Enterobacter cloacae, Pantoea vagans, Lactococcus lactis*, and *Pseudomonas monteilii*, and uncovered some niche division of the microbial associates in the host alimentary tract. This study provides the basic framework for understanding the gut environment and microbial community of the notorious pest weevil *D. abbreviates*.

The Japanese beetle, *Popillia japonica*, was first detected in 1916 and is now established as a destructive invasive pest in North America, where its larvae infest roots of turf and pasture grasses, and its adults damage landscape plants and crop plants (Potter and Held, 2002). Avila-Arias et al. investigated the archaeal communities of the digestive tract of third instar larvae of *P. japonica* and the associated soil environment using shotgun metagenome sequencing, which uncovered that the compositions of archaeal taxa are different according to gut compartments (midgut vs. hindgut) and experimental conditions (field vs. manipulative laboratory studies). More methane metabolism-related taxa and gene sequences were detected in the larval hindgut, suggesting that methanogenesis mainly occurs in the gut compartment of larval *P. japonica*. While the gut archaeome of *P. japonica* was largely similar to the archaeome of the infested soil, at least one methanogenic archaeal taxon, *Methanobrevibacter*, was enriched in the infested soil samples, which may indicate an aspect of environmental impact of *P. japonica* invasion.

The red flour beetle, *Tribolium castaneum*, has been widely used as a model insect in basic research fields such as population biology, evolutionary ecology and molecular genetics (Campbell et al., 2022), and in applied and economical fields, regarded as a notorious pest of stored products (Rees, 2004). Sauce-flavor Daqu is a high-temperature, solid fermentation starter brick made from grains that provides the microbes, enzymes, and flavor precursors needed to brew Chinese sauce-flavor Baijiu, which often suffers deterioration and loss due to infestation by *T. castaneum* during storage. Lü et al. examined bacterial and fungal microbiota of sauce-flavor Daqu and infesting larvae and adults of *T. castaneum* using 16S and ITS amplicon sequencing, which provided microbial basis for understanding how the traditional fermented food product is affected by the pest insect infestation.

In conclusion, the Research Topic provides an overview of recent and novel studies on the beetle-microbe symbiotic associations. In particular, it highlights a variety of microbial associations in economically important beetle species including biocontrol agents, forest pests, agricultural pests, and stored product pests. Considering the outstanding diversity of beetles, which embraces over 400,000 described species and represents the majority of the biodiversity in the terrestrial ecosystem, there is no doubt that many more interesting beetle-microbe symbiotic associations are awaiting discovery and investigation in the future.
